# Imbalance of c-MPL Isoform Promotes Tumorigenesis by Activating STAT-5 in Leukemic Cell Lines

**DOI:** 10.3390/cimb48070713

**Published:** 2026-07-13

**Authors:** Mohammad Amjad Hussain, Mithila Kulkarni, Reginald Samson Valder, Suparna Laha

**Affiliations:** Cell Biology and Molecular Genetics Division, Yenepoya Research Centre, Yenepoya (Deemed to be University), Mangaluru 575018, India; amjadhussain318@gmail.com (M.A.H.); mithilakulkarni68@gmail.com (M.K.); valdarreginald@gmail.com (R.S.V.)

**Keywords:** c-MPL receptor, leukemia, apoptosis, c-MPL-FL/c-MPL-TR ratio, proliferation

## Abstract

Leukemia is a hematopoietic defect, involving complex molecular and cellular alterations, in which dysregulated signaling through hematopoietic receptors, including c-MPL (c-Myeloproliferative Leukemia), has been implicated. The function of c-MPL is mostly regulated by the crosstalk and stoichiometry of its different isoforms. Although expression of c-MPL in hematological disorders has been studied, the regulation of its isoforms and their balance, functional roles, and mechanisms of action in conditions such as acute and chronic leukemia and myeloproliferative neoplasms remain poorly understood. The association of c-MPL isoforms with leukemia cell proliferation and aggressiveness was examined by immunophenotyping, immunofluorescence, RT-PCR, Western blotting and clonogenic assay. This study demonstrates that an increase in the ratio of c-MPL-Full Length (FL)/c-MPL-Truncated (TR), the conserved isoforms of c-MPL, influences tumorigenic markers such as Ki67, Caspase-3, and BCL-2, thereby promoting aggressiveness in leukemic cell lines. Furthermore, we have observed that with an increase in the c- MPL-FL/c-MPL-TR ratio, STAT5 activation increases, promoting the proliferative state of leukemic cells, thereby revealing c-MPL isoforms as a therapeutic target for leukemia. In this work, we observed an increased c-MPL expression in leukemic cell lines, but cell proliferation is independent of total c-MPL expression. Our study demonstrates the regulatory role of c-MPL isoforms, particularly c-MPL-FL, in increasing cell proliferation in leukemia cell lines. This finding is a step towards developing c-MPL isoform as a therapeutic target for leukemic conditions such as acute and chronic leukemias and myeloproliferative neoplasms, but it needs further investigation for complete validation.

## 1. Introduction

**Figure 1:**
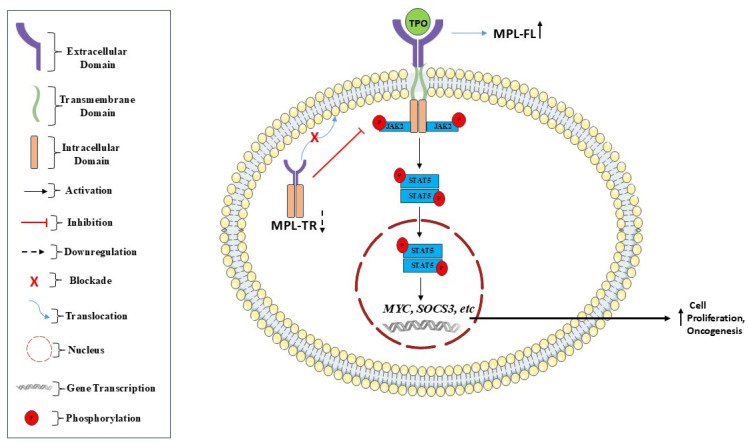
Schematic representation of c-MPL/STAT5 signaling pathway. This figure represents the schematic diagram of STAT5 activation in the presence of c-MPL-FL, leading to increased cell proliferation. All symbols, colors, arrows, phosphorylation marks, and line styles used in the schematic are defined in the figure. The cell and nuclear membrane have been designed using Med Servier Art licensed under Creative Commons Attribution 4.0 International (CC BY 4.0).

Leukemiais a group of hematological cancers arising from the uncontrolled proliferation of abnormal white blood cells. These abnormal, non-functional immune cells deplete the pool of hematopoietic stem cells (HSCs) and occupy space, leading to an improper production of normal white blood cells, platelets, and red blood cells in the bone marrow [1]. Leukemia has been divided into several classes depending on its cell origin and maturity. Myeloid or myelogenous leukemia describes cancers that affect cells derived from myeloid progenitors, such as mast cells, neutrophils, basophils, eosinophils, erythrocytes, and macrophages. On the other hand, lymphoid or lymphoma refers to those that impact lymphocytes, such as T cells, B cells, and natural killer cells. Further classification of acute and chronic leukemia is based on immature hematopoietic cells and partially matured hematopoietic cells, respectively [2]. These defects in the blood are maintained and initiated by a small subset of cells known as leukemia stem cells (LSCs), which can form tumor cells.

c-Myeloproliferative leukemia (c-MPL) is considered the LSC surface marker, making cells resistant to conventional therapy [3]. It is also well known for regulating hematopoiesis and maintaining hematopoietic stem cells (HSCs) and is the key cytokine regulating platelet production from megakaryocytes [4]. Total MPL expression consists of MPL-Full Length (FL), MPL-Truncated (TR), MPL-K, and MPL-del. It is a member of the hematopoietic growth factor receptor superfamily, and encodes the 635-amino-acid protein c-MPL, which has a signal peptide domain, two extracellular cytokine receptor domains, a transmembrane domain, and two intracellular cytokine receptor box motifs [5]. c-MPL-FL is essential for the maintenance of hematopoietic stem cells (HSCs) and serves as the primary regulator of platelet production from megakaryocytes. It is the primary cell-surface receptor required for TPO-mediated activation of the JAK/STAT, MAP/ERK, and PI3K/AKT signaling pathways. A truncated isoform, c-MPL-TR, is generated due to the absence of the transmembrane domain in c-MPL-FL, and both isoforms are evolutionarily conserved across species. The balance between c-MPL-FL and c-MPL-TR regulates TPO-dependent signaling in an isoform-ratio-dependent manner (Figure 1). c-MPL-K arises due to premature termination in the unspliced region of intron 10 and does not respond to TPO-mediated signaling. c-MPL-D, on the other hand, contains 24 amino acids fewer than c-MPL-FL but dimerizes with c-MPL-FL and also promotes TPO binding and internalization, leading to activation of different pathways [6]. Studies investigating total c-MPL expression have identified oncogenic activating mutations and functional abnormalities associated with several hematological malignancies and disorders [7,8,9,10,11]. In addition, even in the absence of mutations, many AML patients overexpress c-MPL, and HSC proliferation and survival are enhanced in response to TPO. Overexpression of total c-MPL correlates with shorter and complete remission in patients with AML [12]. Additionally, patients with different types of leukemia have increased c-MPL expression, which correlates with CD34 expression [13]. Furthermore, patients with c-MPL^+^ LSCs have a worse prognosis and are resistant to conventional chemotherapy [3]. In Rac1-mediated leukemia initiation and maintenance, the overexpression of total c-MPL plays a vital role in the interaction of leukemia cells with the bone marrow (BM) niche and contributes to quiescence and chemotherapy resistance [14]. In a few chronic myeloid leukemia (CML) cases, mutations in the c-MPL genes are also observed [15]. An increase in c-MPL expression indicates greater leukemic properties and resistance to BCR-ABL inhibitors in CML [16]. Expression of c-MPL was also detected in the majority of B cell lines in an in vitro study, suggesting that the TPO/c-MPL interaction might play an important role in the growth and regulation of B-precursor leukemia cells [17]. In acute lymphoblastic leukemia (T-ALL) conditions, c-MPL has been implicated in the self-renewal of both normal and leukemic stem cells, and its expression also contributes to chemo-resistance and disease progression [18]. Collectively, these studies indicate that c-MPL is highly expressed in leukemic conditions, contributing to therapy resistance and maintenance of stem cell-like properties of LSCs.

However, it remains unclear whether all these functions are mediated by total c-MPL or by the relative balance of its conserved isoforms, c-MPL-FL and c-MPL-TR. Therefore, understanding the mechanisms and interplay of these isoforms and contributing to cell proliferation in AML is essential. In this present in vitro study, we investigated the expression ratios of c-MPL-FL/c-MPL-TR and their association with enhanced cell proliferation and STAT5 pathway activation in leukemic cell lines.

## 2. Materials and Methods

### 2.1. Materials

The cell lines K562, KG1, HL60, and Jurkat used in the study represent different hematological malignancies and stages of differentiation, enabling comparative analysis of c-MPL isoform expression across leukemic cell subtypes. The cell lines were primarily procured from NCCS Pune, India, except the PBMC cells, which were purchased from Himedia, Mumbai India. PBMCs were collected from a single healthy donor and used as control cells, and an ethical clearance certificate was also provided by Himedia. The antibodies used in the study were sourced from various sources: MPL and Ki67 from Invitrogen, Waltham, MA, USA; β-actin, STAT, pSTAT, and Caspase-3 from Cell Signalling Technology (CST) Danvers, MA, USA; and CD44+ from Abcam Fremont, CA, USA. Antibody details, including source, catalog number, and working dilution, are provided in Table 1. The c-DNA synthesis kit (RR037A) and SyBr Green (RR820A) were purchased from Takara, Kusatsu, Shiga, Japan. The DAPI used for immunofluorescence, propidium iodide (PI), and RNase A used in cell cycle analysis were all purchased from Himedia, Mumbai, India. Low-melting agarose was also procured from Himedia, Mumbai, India.

### 2.2. Cell Culture

The human leukemic cell lines K562 (CML), KG1 (AML), HL60 (APML), and Jurkat (ALL), and normal cells, PBMCs, were cultured in RPMI-1640 supplemented with 10% fetal bovine serum (FBS), 1 mM sodium pyruvate, and 50 IU/mL antibiotics/antimycotics containing penicillin (100 U/mL), streptomycin (100 μg/mL) and amphotericin B (0.25 μg/mL) (Gibco, Grand Island, NY, USA) and maintained at 37 °C with 5% CO_2_. All the experiments were performed after the cells reached 70–75% confluency.

### 2.3. Preparation of Cell Lysates and Western Blot Analysis

After harvesting, the cells were quickly placed on ice and washed with ice-cold PBS. Subsequently, they were treated with RIPA lysis buffer (20 mM Tris-HCl (pH 7.4), 150 mM NaCl, 5 mM EDTA, 1% Nonidet P-40, 1 mM Na3VO4) supplemented with proteinase inhibitor (Cat. No ML051 Himedia) [19]. Sonication was performed for 3 min at 30 amps with a 10 sec on/off pulse. Protein estimation was performed with Bradford’s reagent. Equal amounts of protein (50 µg per lane) were loaded for all samples to ensure uniform comparison across cell lines. Chemiluminescent and gel images were captured and analyzed using a Fusion Solo Chemi/Gel documentation system (Vilber Lourmat, Collégien, France). The quantification of the band intensities was performed using the Fusion Capt Gel doc software v18-02. Expression values were calculated relative to the HL60 c-MPL-FL band, which was assigned a value of 1.0. All other bands, including TR, were normalized to the assigned value for HL60 to improve comparability. β-actin was used as an internal loading control for normalization of protein expression, which was used for both c-MPL-FL and TR calculation, followed by analyzing the c-MPL-FL/TR ratio. For STAT-5 and pSTAT-5, HL60 was used as the calibrator sample and assigned a value of 1.0. Expression in other cell lines is shown relative to HL60, and densitometric values represent relative protein expression normalized to β-actin. All Western blot experiments were performed using a minimum of three independent biological replicates (*n* = 3).

### 2.4. RNA Extraction and RT–PCR

RNA extraction was performed using the TRIzol chloroform method, and the RNA was quantified using a micro-volume Titertek Berthhold spectrometer [20]. Reverse transcription for c-DNA synthesis was performed using 1 µg of total RNA in a final volume of 20 µL and incubated at 37 °C for 15 min, followed by 85 °C for 5 s as per the data sheet. RT–PCR was performed using 100 ng of cDNA in a final volume of 10 µL containing 2× SYBR Green master mix and 10 µM of forward and reverse primers for the desired gene, *c-MPL*, *β-actin*, *Bcl-2* or *TPO*. Gene expression levels were normalized using *β-actin* as a housekeeping gene. The sequence for all the primers used in this study are summarized in Table 2. RT-PCR experiments were performed with technical triplicates of each sample in three independent biological replicates (*n* = 3).

### 2.5. Cell Cycle Analysis

The cells were seeded overnight in a 60 mm dish at a density of 1 × 10^6^ cells per dish for each cell line. After incubation, cells were harvested, and approximately 0.5 million cells were collected in an Eppendorf tube and washed with PBS. The cells were then fixed in ethanol at −20 °C for 1 h. After washing with PBS, the cells were incubated in a DNA staining solution containing 0.5 mg/ml PI, 0.1% sodium citrate, 2% Triton X-100, and 2 mg/mL RNase A for 20 min in the dark [21]. Cell cycle analysis was then performed using Guava flow cytometry (Merck, Millipore, FCS version 7.0). Cell cycle experiments were performed in three independent biological replicates (*n* = 3).

### 2.6. Immunofluorescence

The expression of the cell surface marker c-MPL was analyzed by immunofluorescence using the following protocol: 0.5 × 10^6^ cells were washed with PBS, then fixed with 4% PFA (paraformaldehyde) and incubated at room temperature in the dark for 20 min. The supernatant was discarded after centrifugation, and the cells were then treated with a permeabilization agent (0.1% Triton X-100 in PBS) and incubated at RT for 15 min, followed by PBS wash. Blocking was performed with 2% BSA in PBS for 1 h. Primary antibody incubation was performed overnight at 4 °C in 0.1% BSA in PBS. After washing, the cells were incubated with a fluorescently tagged secondary Alexa Fluor 488 antibody in PBS for 1 h. The cells were then stained with DAPI after a PBS wash, incubated at room temperature for 20 min, mounted on a slide and visualized by ZOE Fluorescent Cell Imager Bio Rad. These experiments were performed in three independent biological replicates (*n* = 3).

### 2.7. Immunophenotyping

A total of 0.5 × 10^6^ cells were collected and washed with PBS, followed by 15 min of incubation in FACS staining buffer containing 2% (*v*/*v*) FBS, 96% (*v*/*v*) PBS, 1% (*v*/*v*), 0.5 M EDTA, and 1% (*v*/*v*) penicillin/streptomycin used for flow cytometry analysis. The supernatant was discarded after centrifugation, and the cells were then treated with a permeabilizing agent (0.1% Triton X-100 in PBS) and incubated at RT for 15 min, followed by PBS washes. Blocking was performed with 2% BSA in PBS for 1 h. The cells were then incubated in FACS staining buffer, followed by incubation with the primary antibody at 4 °C overnight. Then, the cells were incubated with Alexa Fluor 488, a fluorescent-tagged secondary antibody, for 1 h at RT. The cells were then washed, and fresh FACS buffer was added. Unstained cells were used as negative controls to establish gating parameters and background fluorescence during flow cytometric analysis. The data were acquired through Guava flow cytometry (Merck, Millipore, Temecula, CA, USA, FCS version 7.0). Immunophenotyping experiments were performed in three independent biological replicates (*n* = 3).

### 2.8. Clonogenic Assay

The clonogenic assay was carried out following the Borowicz et al. 1998 protocol [22]. Briefly, to grow suspension cell colonies, a base layer was prepared by mixing equal volumes of 1% low-melting agarose with complete RPIM-1640 media, which was kept at 42 °C. This was poured into a 6-well plate and allowed to solidify at room temperature for 10 min. The top layer was prepared by suspending the cells (*n* = 10,000) in pre-warmed complete media and mixing this 1:1 with 0.6% low-melting agarose. Plates were kept at room temperature for 5–10 min until agarose completely solidified. Above the agarose, 1.5 mL of media was added, and the plates were transferred to the incubator, maintained at 37 °C with 5% CO_2_. Fresh media was added twice a week to keep the cells healthy. Colonies were allowed to develop for 10 days, after which they were visualized by staining with crystal violet and counted using ImageJ software, version 1.54d (National Institutes of Health, Bethesda, MD, USA). The clonogenic assay was independently performed twice (*n* = 2), as biological replicates.

### 2.9. Acridine Orange/Ethidium Bromide Staining

The apoptotic morphology of the cells and the quantity were evaluated using acridine orange (AO)/ethidium bromide (EB). Briefly, 0.5 × 10^6^ cells were collected and centrifuged at 1200 rpm for 2 min. The cell pellet was washed once with PBS. A working stain solution was prepared by mixing an equal volume of AO and EB (100 µg/mL). Subsequently, 10 µL of suspension was mixed with 2 µL of AO/EB staining solution and gently mixed. The stained cell suspension was immediately placed onto a clean glass slide, covered with a coverslip, and observed under a fluorescent microscope. Cell viability was assessed based on fluorescence characteristics, where viable cells exhibited a uniform green nucleus and apoptotic cells showed an orange-colored appearance [23].

### 2.10. Statistical Analysis

All experimental data are presented as mean ± standard error of the mean (SEM) and were obtained from independent biological replicates, as specified for each experiment. Statistical analysis was performed using GraphPad Prism version 8.4.3 (GraphPad Software, San Diego, CA, USA). Comparisons among groups were conducted using one-way analysis of variance (ANOVA) followed by Tukey’s post hoc multiple comparisons test, as appropriate, to identify statistically significant differences. A *p*-value of less than 0.05 was considered statistically significant.

## 3. Results

### 3.1. Total c-MPL Expression Is Higher in Different Leukemic Cell Lines Compared to Normal Cells

**Figure 2:**
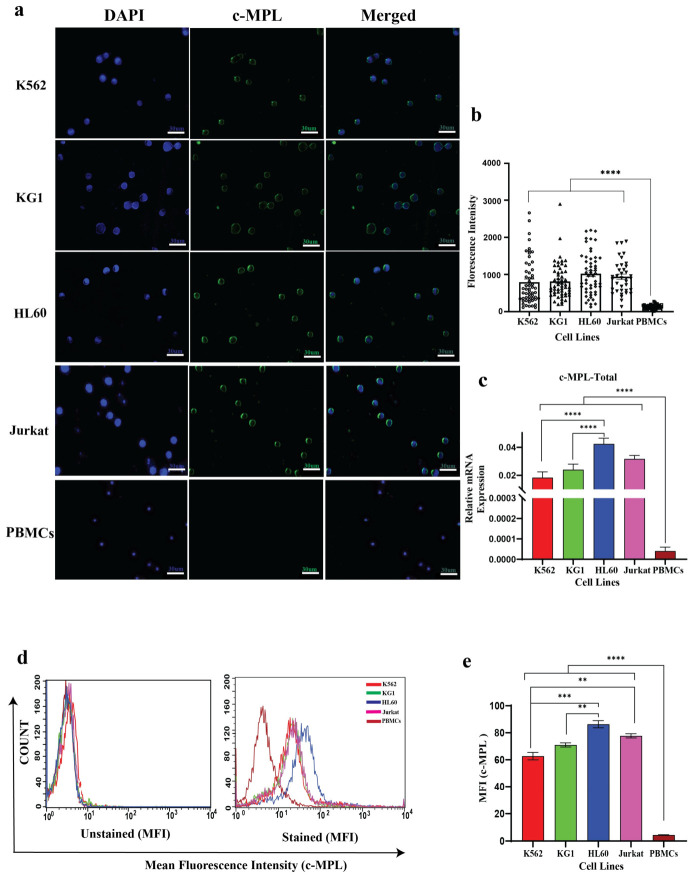
Total c-MPL is expressed more in various leukemic cell lines than in the control line. (**a**) Immunofluorescence of c-MPL across different cell lines. The green color (center panel) represents c-MPL, blue (first panel) represents DAPI, and the third panel is the merged image of DAPI and c-MPL. (**b**) Quantitative analysis of c-MPL fluorescence intensity. (**c**) Relative total c-MPL mRNA expression across different cell lines assessed by RT-PCR. (**d**,**e**) Histogram represents flow cytometry analysis of total c-MPL in K562 (red), KG1 (green), HL60 (blue), Jurkat (pink/magenta), and PBMC (brown). The x-axis and the y-axis in the histogram represent the mean fluorescence intensity (MFI) and the cell count, respectively. and corresponding bar graphs showing c-MPL expression in unstained and stained (with c-MPL antibody) samples across different cell lines. Data are presented as mean ± SEM from three independent biological triplicates (*n* = 3) for each experiment shown. Statistical analysis was performed using one-way ANOVA followed by Tukey’s multiple comparison test. Statistical significance is represented as ** *p* ˂ 0.01, *** *p* ˂ 0.001, **** *p* ˂ 0.0001 between different cell lines.

The c-MPL receptor maintains the proliferative and quiescent state of HSCs [24]. To understand the correlation between leukemia and c-MPL, we examined the relative expression of c-MPL in various leukemia cell lines and normal PBMCs. As both myeloid and lymphoid cells originate from the common progenitor cells, i.e., HSC, we performed in vitro analysis using different leukemic cell lines, including KG1 (acute myeloid leukemia—AML), K-562 (chronic myeloid leukemia—CML), HL-60 (acute promyelocytic leukemia—APL), a subtype of AML, and Jurkat (T cell acute lymphoblastic leukemia—T-ALL), along with peripheral blood mononuclear cells (PBMCs). PBMCs were used as a non-malignant hematopoietic reference population to compare relative c-MPL expression in leukemic cell lines; however, they may not fully represent normal hematopoietic stem cell physiology, which remains a limitation of this work [25,26]. Our findings revealed that all leukemia cell lines expressed significantly higher levels of c-MPL than the control cells. Among these, the HL60 and Jurkat cell lines, which reflect the M2 stage of AML in the FAB classification [27] and T-ALL, respectively, showed the greatest amount of c-MPL expression. Immunofluorescence assays showed c-MPL proteins on the cell membrane, with stronger fluorescence signals in leukemia cell lines than in PBMCs (Figure 2a,b). Through qRT-PCR analysis, we further found that, compared with normal cells, all the leukemia cell lines expressed higher levels of total c-MPL. Furthermore, total c-MPL expression in HL60 and Jurkat cells was higher than in the other leukemia lines, KG1 and K562 (Figure 2c). Similar results were obtained by analyzing total c-MPL expression through immunophenotyping in different cell lines (Figure 2d,e). Collectively, these results validated the enhanced expression of total c-MPL in leukemia cell lines. Also, there is variation in c-MPL overexpression across the leukemia lines, particularly in the HL60 and Jurkat cell lines, providing a clue to the interplay of other factors in leukemic pathophysiology.

### 3.2. The Ratio of c-MPL-FL/TR Isoforms Varies Within the Leukemia Cell Lines

**Figure 3:**
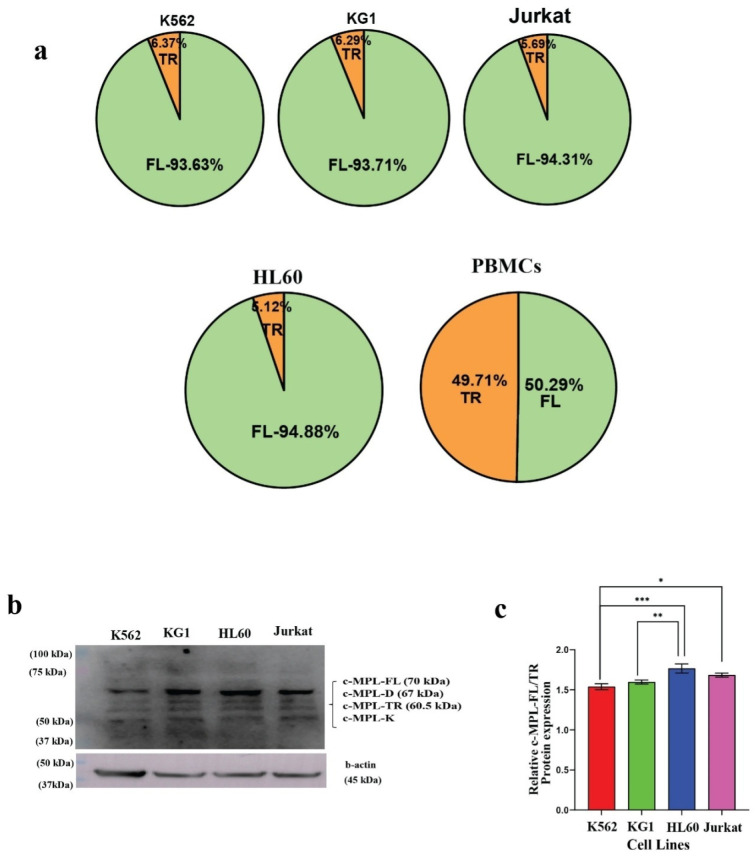
Increase in c-MPL-FL/c-MPL-TR ratio in certain leukemia cell lines. (**a**) Pie chart representation of RT-PCR data showing the relative expression in percentage of c-MPL-FL and TR isoforms. Green sectors represent MPL-FL, whereas orange sectors represent MPL-TR. Values within the pie charts indicate the percentage of each isoform (**b**) Western blot analysis of c-MPL isoform across different cell lines: K562 (red), KG1 (green), HL60 (blue), and Jurkat (pink/magenta). The molecular weight of the different isoforms ranges from 60 kDa to 80 kDa. (**c**) Densitometric analysis of Western blot bands using Chemi Doc software v18.2, normalized to β-actin. Data are presented as mean ± SEM from three independent biological triplicates (*n* = 3) for each experiment shown. Statistical analysis was performed using one-way ANOVA followed by Tukey’s multiple comparisons test. Statistical significance is represented as * *p* ˂ 0.05, ** *p* ˂ 0.01, *** *p* ˂ 0.001, between different cell lines.

In the above section, although we observed increased total c-MPL expression in all leukemic cell lines compared to normal cell lines, the expression varies between the cell lines. To explain this variation, we examined the expression of isoforms and their ratio profiles using RT-PCR. Previous studies have reported that there are at least four different isoforms of c-MPL, with c-MPL FL and TR conserved across species, and a stoichiometric balance between c-MPL-FL and c-MPL-TR isoforms plays a critical role in regulating hematopoiesis, i.e., the higher the level of the c-MPL-FL isoform, the greater the proliferation of the hematopoietic stem cells and hence the more pronounced the tumorigenic condition [28]. We observed that even though the expression of c-MPL and its isoforms was low in the normal PBMCs, the balance between the two isoforms was properly maintained, as shown in the pie chart analysis (Figure 3a). Surprisingly, the leukemic cell lines (K562, KG1, HL60 and Jurkat) showed a completely compromised balance between the two isoforms. The expression analysis demonstrated that the FL variant was higher than the TR isoform across all cell lines (Figure 3a). Upon analysis of the ratio of the c-MPL-FL/TR isoforms, PBMCs showed a lower c-MPL FL/TR ratio. In comparison, the ratio of the two isoforms was significantly higher in the HL60 cells compared to the other leukemia cells, K562, KG1 and Jurkat. These results showed that c-MPL expression, particularly c-MPL-FL transcripts, is higher in the HL60 cell line compared to other leukemia cell lines. A comparative Western blotting experiment further revealed that the c-MPL/TR ratio was higher in the HL60 and Jurkat cell lines than in other leukemic cell lines (Figure 3b,c), confirming the RT-PCR results and suggesting that the c-MPL-FL/TR isoform ratio may contribute to the variation in tumorigenic properties between the different leukemic conditions.

### 3.3. The Imbalance in the c-MPL-FL/TR Ratio Promotes the Tumorigenic Properties in Leukemia Cell Lines

**Figure 4:**
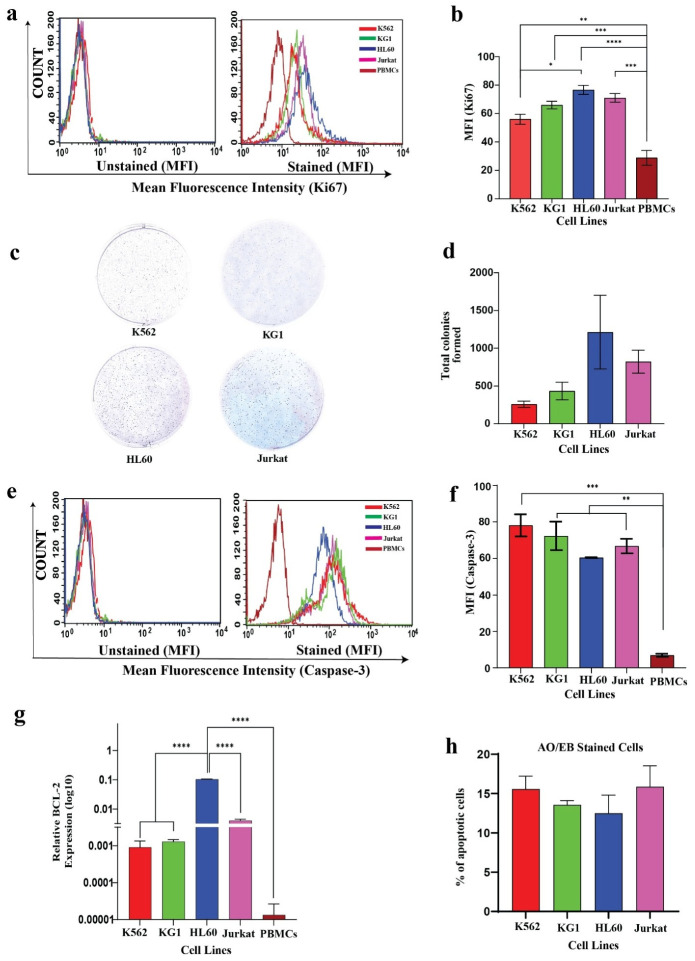
The differential tumorigenic potential of leukemic cell lines is associated with the increase in the ratio of c-MPL-FL/c-MPL-TR. (**a**) Flow cytometry histograms and corresponding bar graphs showing Ki67 expression across different cell lines, in unstained and stained samples (*n* = 3). Histogram overlays represent K562 (red), KG1 (green), HL60 (blue), Jurkat (pink/magenta), and PBMCs (brown) (**b**) Quantitative analysis of Ki67 expression across different cell lines through immunophenotyping. (**c**,**d**) Clonogenic assay and corresponding analysis give the functional validation of the proliferative potential (*n* = 2). (**e**,**f**) Flow cytometry histograms and corresponding bar graphs showing Caspase-3 expression across different cell lines, in unstained and stained samples (*n* = 3). Histogram overlays represent K562 (red), KG1 (green), HL60 (blue), Jurkat (pink/magenta), and PBMCs (brown). The x-axis and the y-axis in histogram represent the mean fluorescence intensity (MFI) and the cell count, respectively (**g**) The bar graph represents the Bcl-2 relative mRNA expression in leukemia and normal cell lines through RT-PCR (*n* = 3). (**h**) The functional validation of the apoptotic potential of the leukemic cells. Microscopic fields with a total of 150 cells were analyzed. The bar graph represents % apoptotic cells. Statistical analysis was performed using one-way ANOVA followed by Tukey’s multiple comparisons test. Statistical significance is represented as * *p* ˂ 0.05, ** *p* ˂ 0.01, *** *p* ˂ 0.001, **** *p* ˂ 0.0001 between different cell lines.

After confirming an increase in the c-MPL FL/TR ratio in various leukemia lines compared with normal PBMCs, with the highest in the HL60 cell line, we next sought to understand the relationship between the ratio of c-MPL isoforms and cellular proliferation. We examined the cell cycle patterns in different leukemic cell lines. We found that most leukemic cells are in the G1 and G2/M phases of the cell cycle and spend less time in the S-phase. This observed cell cycle distribution suggests ongoing cell cycle activity and may reflect the proliferative characteristics of the leukemic cell lines (Supplementary Figure S1A,B). We confirmed the proliferative nature of these cell lines by assessing Ki-67 protein expression, a well-established marker for cell proliferation. All leukemia cell lines showed higher Ki-67 expression than the normal cell line (PBMC). Among the leukemia cell lines, the HL60 cells, which have the highest c-MPL FL/TR ratio, showed the highest Ki67 expression, followed by Jurkat cells, indicating a more proliferative and aggressive phenotype (Figure 4a,b). To further assess the proliferative capacity and aggressive growth characteristics of the HL60 cell line compared to the other cell lines, we performed a soft agar clonogenic assay to evaluate colony formation ability and anchorage-independent growth, which are associated with enhanced cellular aggressiveness [22]. We found that among all cancerous cell lines, HL60 and Jurkat showed the highest colony formation after 10 days of cell seeding (Figure 4c,d). We also evaluated Caspase-3 expression to assess the differential apoptotic activity among the leukemia cell lines. Caspase-3 is an apoptotic marker whose expression decreases in severe AML cases [29]. We observed that fewer cells in the HL60 cell line exhibited Caspase-3 expression than the other leukemia cell lines, indicating reduced apoptotic activity in HL60 cells. This decreased apoptosis may contribute to increased cellular proliferation and lead to the accumulation of tumor cells (Figure 4e,f). We also evaluated BCL-2 expression in leukemia cell lines and PBMCs. BCL-2 acts as an anti-apoptotic protein by stabilizing mitochondrial membrane integrity and preventing the release of cytochrome c, which otherwise leads to apoptosis [30]. It has also been reported that BCL-2 overexpression is associated with chemoresistance in AML cases, making this gene a therapeutic target [31]. We found that BCL-2 expression was higher in leukemic cases than in PBMCs, with HL60 and Jurkat showing even higher expression, indicating a more aggressive phenotype (Figure 4g). Finally, the functional validation of apoptosis was carried out by AO-EB staining which further confirmed that the HL60 cell line had most proliferative cells as it had the fewestapoptotic cells (Figure 4h). The representative field images are given as Supplementary Figure S2. The increased proliferative potential was further supported by the expression of the tumorigenic/stem cell marker CD44^+^ in different cell lines. Notably, we observed that the HL60 cell line had a notably higher proportion of CD44^+^ cells than the other leukemia cell lines, again indicating the aggressiveness of the HL60 cell line (Supplementary Figure S3a,b). Therefore, despite all the leukemia lines showing high levels of c-MPL expression, we observed variability in the proliferative behavior, as evidenced by differences in stemness, apoptotic resistance, and proliferation markers. The HL60 cell line showed the highest expression of these markers (Ki67, CD44+, and Bcl-2) compared to the other leukemia cell lines. These findings indicate that the ratio of c-MPL-FL/TR is one of the key factors regulating proliferation and tumor aggressiveness.

### 3.4. Overexpression of c-MPL-FL Exacerbates Leukemic Cell Proliferation Through Activation of the STAT Pathway

**Figure 5:**
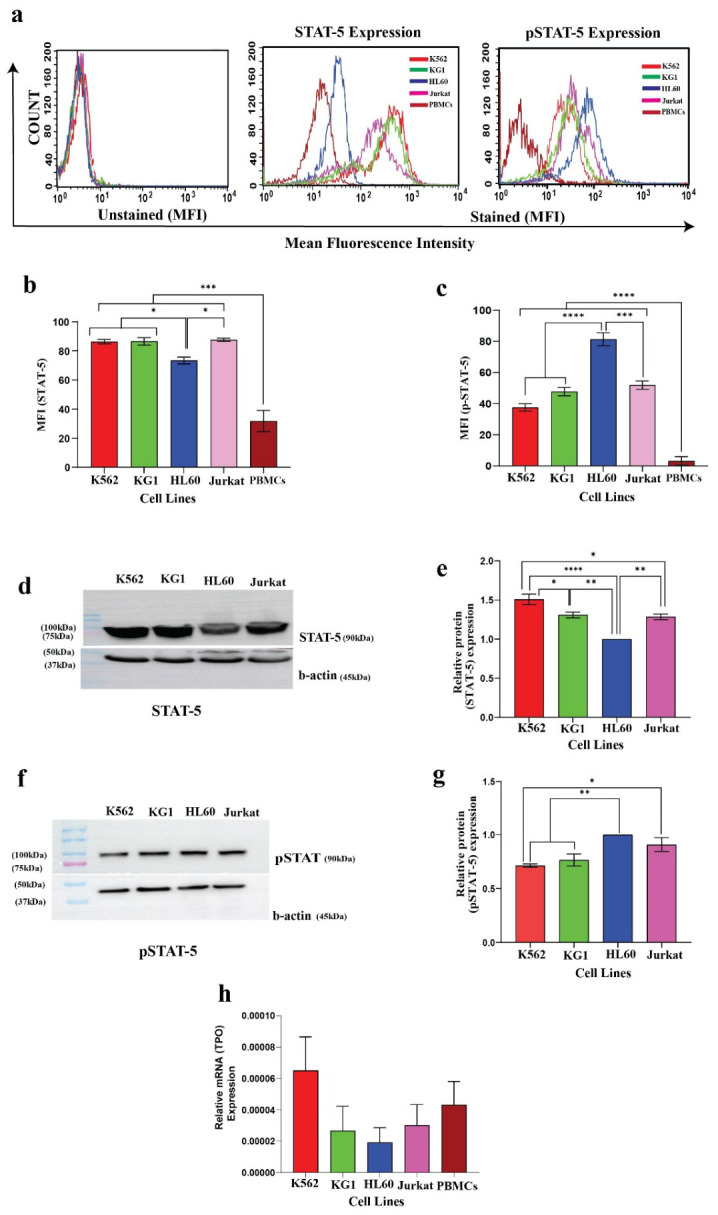
Overexpression of c-MPL-FL is associated with increased STAT5 activation. (**a**) Flow cytometry histograms showing STAT5 and pSTAT5 expression in unstained and stained samples across different cell lines. Histogram overlays represent K562 (red), KG1 (green), HL60 (blue), Jurkat (pink/magenta), and PBMCs (brown). The x-axis and the y-axis in the histogram represent the mean fluorescence intensity (MFI) and the cell count, respectively. (**b**,**c**) Quantitative expression of STA5 and pSTAT5 by immunophenotyping and represented as MFI values. (**d**,**e**) Western blot and densitometric quantification of total STAT5 in K562, KG1, HL60, and Jurkat cells. β-actin was used as the loading control. (**f**,**g**) Western blot and corresponding quantification of phosphorylated STAT5 (pSTAT5) across different cell linesin K562, KG1, HL60, and Jurkat cells. β-actin was used as the loading control. (**h**) Relative *TPO* mRNA expression in leukemic cell lines and PBMCs determined by RT-PCR. Bar colors correspond to K562 (red), KG1 (green), HL60 (blue), Jurkat (pink/magenta), and PBMCs (dark red/brown). All data are presented as mean ± SEM from a minimum of three independent biological triplicates (*n* = 3) for each experiment shown. Statistical analysis was performed using one-way ANOVA followed by Tukey’s multiple comparisons test. Statistical significance is represented as * *p* ˂ 0.05, ** *p* ˂ 0.01, *** *p* ˂ 0.001, **** *p* ˂ 0.0001 between different cell lines.

In this section, we investigated the possible mechanism underlying enhanced cell proliferation and tumorigenesis driven by the increased c-MPL-FL/TR isoform ratio. Since STAT5, a critical downstream molecule of c-MPL signaling, is activated following the dimerization of c-MPL-FL receptor, its phosphorylated form (pSTAT5) acts as a transcription factor and regulates the expression of genes that promote cell proliferation and survival [32]. As the c-MPL-TR isoform lacks the transmembrane domain, it is unable to localize to the cell membrane, resulting in reduced functional c-MPL receptor expression and consequent downregulation of TPO-mediated proliferation of HSCs. This absence of TPO/c-MPL-TR interaction inhibits TPO internalization, thereby preventing downstream activation. In some cases, constitutive activation of c-MPL happens in the absence of TPO binding due to mutations in the extracellular or cytoplasmic juxtamembrane regions. Because of the absence of both the juxtamembrane and transmembrane regions, the TR isoform is capable of regulating both the TPO-dependent and TPO-independent ways of activation of c-MPL (Figure 1). To study the activation of the downstream pathways by overexpressed c-MPL-FL isoforms in HL60 and Jurkat cells, we examined their effect on downstream effector molecules through immunophenotyping and Western blotting experiments. We found that, compared with normal cells (PBMCs), the levels of STAT5 in the leukemia cell lines were greater (Figure 5a). While the inactive STAT5 levels were relatively low in HL60 cells, the levels of pSTAT5 were significantly higher in this cell line compared to the other leukemic cell lines (Figure 5b,c). The activation of STAT5 was further confirmed by Western blot analysis. The intensity of the STAT5 band in the Western blot was significantly lower in HL60 cells compared to the other leukemic cells (Figure 5d,e), whereas the phosphorylated STAT5 expression was significantly higher in the HL60 line (Figure 5f,g). To confirm that the STAT5 activation is directly proportional to the c-MPL-FL isoform when it is overexpressed and is independent of the availability of TPO, we analyzed the TPO expression in all the leukemic cell lines compared to the non-leukemic control cells. Surprisingly, we observed no significant difference in the TPO expression, whereas there was a significant difference inSTAT5 activation in the cells with high c-MPL-FL expression compared to the control cells (Figure 5h). This indicates that cells with higher c-MPL-FL isoforms, such as HL60 and Jurkat, activate STAT5 more, leading to stronger proliferative and transcriptional signals, which in turn contribute to the increased tumor aggressiveness by increasing cell proliferation. Through this study, we found a strong correlation between c-MPL-FL and STAT5 activation, which was associated with increased cellular proliferation in leukemia cell lines. Both inactive and phosphorylated STAT levels were low in normal cells, suggesting that the STAT5 activation is linked to the proliferation of cancerous cells.

## 4. Discussion

The present study examines the role of c-MPL isoforms, rather than the total c-MPL, in modulating the proliferation and aggressive behavior of leukemia cells. c-MPL is a key regulator of hematopoietic stem cell maintenance and megakaryopoiesis. Irregularities in c-MPL have been found in various malignant hematological disorders. Although increased c-MPL expression has previously been associated with leukemia progression, the contribution of specific c-MPL isoforms remains poorly understood. Our findings suggest that alteration in the c-MPL-FL/TR ratio, rather than total c-MPL abundance alone, may contribute to leukemic progression and its disease aggressiveness.

In our study, we found that although all leukemic cell lines exhibited higher c-MPL expression than PBMCs, we observed significant variation in c-MPL overexpression within leukemic cell lines. This observation suggests that, in addition to total c-MPL, other factors may play an important role in the development of leukemia in these cells (Figure 2). We further evaluated the expression of c-MPL isoforms in different leukemic cell lines and PBMCs. Notably, the predominance of c-MPL-FL/TR isoform ratio expression was elevated in all leukemic cell lines, particularly in HL60 cells, suggesting that altered splicing of c-MPL, i.e., c-MPL-TR formation, may favor downregulation of constitutive signaling activation (Figure 3). In contrast, PBMCs maintained a balanced c-MPL-FL/TR ratio despite relatively low c-MPL expression, suggesting the preservation of isoform stoichiometry may be important for maintaining normal hematopoiesis homeostasis. The association between a higher c-MPL-FL/TR ratio and increased expression of proliferative and tumorigenic markers further support the biological importance of c-MPL isoform imbalance in leukemia cell lines. Increased expression of markers such as Ki67, Caspase-3, and BCL-2 in leukemic cell lines with elevated c-MPL-FL/TR ratios may indicate enhanced proliferative capacity. Following the proliferation analyses, we also observed that, although there is high expression of total c-MPL in all leukemia cell lines, the levels of cellular proliferation and aggressiveness markers increased with increasing c-MPL-FL/TR ratio isoform expression (Figure 4). The maintenance of baseline TPO expression in all the cell lines further supports the statement that overexpression of c-MPL-FL promotes the proliferative and tumorigenic nature of the cells independent of TPO expression (Figure 5). These findings suggest that the relative abundance of c-MPL-FL/TR ratio isoform may be a stronger determinant of disease progression than total c-MPL.

Previous studies have shown that regulating the two c-MPL isomers is important for megakaryocyte development and hematopoiesis [33]. A knockout model of the spliceosome-associated Ott1 gene demonstrated that Ott1 binds to c-MPL pre-mRNA and regulates splicing between c-MPL-TR and c-MPL-FL in HSCs by regulating epigenetic marks. The activation of STAT5, a critical downstream effector of the TPO/MPL axis, is dramatically reduced in c-MPL-stimulated Ott1-/-HSCs. HSCs expressing a greater ratio of c-MPL-TR/c-MPL-FL have dramatically reduced engraftment when transplanted [34]. Our work also suggests that tumorigenic markers (Ki67, CD44, BCL-2) increase in leukemic conditions, with an increase in the ratio of c-MPL-FL/TR isoform (Figure 2, Figure 3 and Figure 4). Moreover, an increase in the expression of c-MPL-FL isoform disturbs the stoichiometric balance of c-MPL-FL and c-MPL-TR on HSCs, leading to a more proliferative state [35]. These findings support the concept that disruption of c-MPL isoform balance may influence hematopoietic function and potentially contribute to disease transformation.

Mechanistically, high c-MPL-FL expression leads to increased c-MPL dimerization, which internalizes more TPO and activates the JAK-STAT pathway, promoting random proliferation of HSCs and resulting in more severe disease manifestation [35]. The elevated pSTAT5 expression in the leukemic cell line due to an increase in the ratio of c-MPL-FL/TR expression, even in the absence of changes in thrombopoietin (TPO) levels, suggests the possibility of constitutive activation of c-MPL-mediated signaling (Figure 5). Since c-MPL activation typically requires binding of ligand, these findings indicate that increased c-MPL-FL receptor abundance facilitates spontaneous receptor dimerization, resulting in continuous activation of downstream signaling and enhancing cell proliferation; however, further mechanistic validation is required.

On the contrary, c-MPL-TR may exert a regulatory role by maintaining hematopoietic stem cells in a quiescent state. Previous studies suggest that c-MPL-TR may help to preserve HSCs in the G0 phase, thereby preventing excessive proliferation and exhaustion of the stem cell pool [34]. Moreover, since c-MPL-TR and c-MPL-FL are conserved across species, this further underscores their role in hematopoiesis [36]. Thus, disruption of the physiological balance between c-MPL-FL and c-MPL-TR may shift hematopoietic cells toward a more proliferative phenotype, increasing susceptibility to leukemic transformation. Taken together, our findings support a potential association between the c-MPL-FL/TR ratio and leukemic cell proliferation.

## 5. Conclusions

Through this work, we concluded that, in the case of malignancies that are caused by hematopoietic defects, the balance between the two isoforms is disrupted. This loss of stoichiometric balance between c-MPL-FL and c-MPL-TR may drive the uncontrolled cell proliferation characteristic of leukemia, making c-MPL-FL a critical target for understanding the disease mechanisms and potentially developing targeted therapies. However, this study has some limitations. The experiments were performed in leukemia cell lines and did not include AML patient samples. Furthermore, PBMCs were used as non-malignant control; however, PBMCs represent a heterogeneous cell population, and are not fully equivalent to leukemic cell lines or true hematopoietic stem/progenitor cells. Therefore, the findings should be interpreted with this limitation in mind. Further studies incorporating CD34+ stem/progenitor cells or normal hematopoietic progenitor models would provide a more physiologically relevant comparison and further strengthen the translational significance of these findings. In addition, gene knockdown-related studies are required to directly assess the role of c-MPL-FL in conferring tumorigenicity. Therefore, future studies focusing on isoform-specific genetic manipulation, in vivo studies, and large-scale clinical validation of c-MPL isoform ratios are also required. Further investigations involving antisense oligonucleotide-mediated splice modulation or splice switching may provide a rational framework for developing targeted and precision-based interventions for leukemia.

## Figures and Tables

**Table 1:** List of antibodies and their concentrations mentioned in the paper.

Sl. No.	Antibody/ Catalog No	Purpose	Concentration	Company	Secondary Antibody Dilution	Expected Molecular Weight (kDa)
1.	c-MPL(1H2)	Western blotting	1:1000	Invitrogen	1:2000	60–80
2.	STAT5 (D3N2B)	Western blotting	1:1000	CST	1:2000	90
3.	p-STAT5 (D47E7)	Western blotting	1:1000	CST	1:2000	90
4.	c-MPL	Immunofluorescence	1:200	Invitrogen	1:250	
5.	c-MPL	Immunophenotyping	1:200	Invitrogen	1:250	
6.	Caspase-3 (9662)	Immunophenotyping	1:200	CST	1:250	
7.	STAT	Immunophenotyping	1:200	CST	1:250	
8.	p-STAT	Immunophenotyping	1:200	CST	1:250	
9.	CD44 (ab46793)	Immunophenotyping	1:100	Abcam	Conjugated	
10.	Ki67 (PA5-16785)	Immunophenotyping	1:200	Invitrogen	1:250	
11.	β-actin (13E5)	Western blotting	1:1000	CST	1:2000	42–45

**Table 2:** List of primers and their sequences mentioned in the paper.

Primers	Position	Sequence
c-MPL-FL (MPL-P)	Forward primer	5′ GCGATCTCGCTACCGTTTAC 3′
	Reverse primer	5′ AGGAAACTGCCACCTCAGC 3′
c-MPL-TR (MPL-S)	Forward primer	5′ AGGACTGGAAGGAGAC 3′
	Reverse primer	5′ TCAGGCTGCAGTGTCCTAAG 3′
c-MPL-Total	Forward primer	5′ GAGAAGCTTCAGCTCTGAC 3′
	Reverse primer	5′ CAAGTGCCACTGCATCTCCA 3′
Beta-actin	Forward primer	5′ACTGGAACGGTGAAGGTGAC 3′
	Reverse primer	5′AGAGAAGTGGGGTGGCTTTT3′
TPO	Forward primer	5′CGTATGACCTGCTGCTGTGGA3′
	Reverse primer	5′GGGTGAAGAATCTATCCGGGTGG3′
BCL-2	Forward primer	5′GACTGAGTACCTGAACCGGC 3′
	Reverse primer	5′GTTCCACAAAGGCATCCCAGC 3′

## Data Availability

The data supporting this study’s findings will be available upon reasonable request.

## Supplementary Materials

The following supporting information can be downloaded at: https://www.mdpi.com/article/10.3390/cimb48070713/s1.

## Abbreviations

AML	Acute Myeloid Leukemia
AMKL	Acute Megakaryoblastic Leukemia
ANOVA	Analysis of Variance
AO	Acridine Orange
APML	Acute Promyelocytic Leukemia
BCL-2	B-cell Lymphoma 2
BM	Bone Marrow
BSA	Bovine Serum Albumin
CD44	Cluster of Differentiation 44
c-MPL	Myeloproliferative Leukemia
CML	Chronic Myeloid Leukemia
CST	Cell Signaling Technology
DAPI	4′,6-diamidino-2-phenylindole
EB	Ethidium Bromide
EDTA	Ethylenediaminetetraacetic Acid
ERK	Extracellular Signal-Regulated Kinase
FAB	French-American-British Classification
FACS	Fluorescence-Activated Cell Sorting
FBS	Fetal Bovine Serum
HSCs	Hematopoietic Stem Cells
ICMR	Indian Council of Medical Research
JAK	Janus Kinase
Ki-67	Proliferation Marker Protein
LSCs	Leukemia Stem Cells
MAPK	Mitogen-Activated Protein Kinase
MPL-D	Deleted c-MPL Isoform
MPL-FL	Full-Length c-MPL Isoform
MPL-K	c-MPL Isoform K
MPL-TR	Truncated c-MPL Isoform
NCCS	National Centre for Cell Science
PBS	Phosphate Buffered Saline
PBMCs	Peripheral Blood Mononuclear Cells
PFA	Paraformaldehyde
PI	Propidium Iodide
PI3K/AKT	Phosphoinositide 3-Kinase/Protein Kinase B
qRT-PCR	Quantitative Reverse Transcription Polymerase Chain Reaction
RIPA	Radioimmunoprecipitation Assay Buffer
RPMI	Roswell Park Memorial Institute Medium
RT-PCR	Reverse Transcription Polymerase Chain Reaction
SEM	Standard Error of the Mean
STAT5	Signal Transducer and Activator of Transcription 5
SYBR Green	Fluorescent Dye Used in qPCR
T-ALL	T-cell Acute Lymphoblastic Leukemia
TPO	Thrombopoietin
