# Supplementary material for: Imbalance of c-MPL Isoform Promotes Tumorigenesis by Activating STAT-5 in Leukemic Cell Lines
Source: Curr Issues Mol Biol. 2026 Jul 13;48(7):713. doi: 10.3390/cimb48070713 (PMC13408313; doi:10.3390/cimb48070713)
Supplement: Supplementary file 1 [file cimb-48-00713-s001.zip › cimb-4292951-supplementary.pdf]

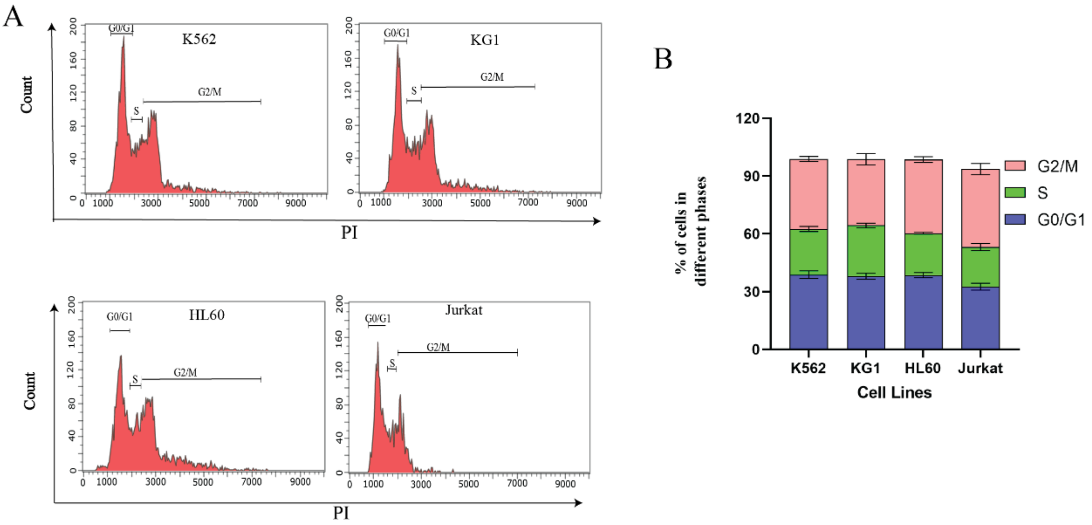

Figure S1: Cell cycle analysis of leukemic cell lines. (A) Representative flow cytometric histograms showing the cell cycle distribution of leukemic cell lines (K562, KG1, HL60, and Jurkat) based on propidium iodide (PI) staining. Cell populations were analyzed for their distribution across the cell cycle, including G0/G1, S, and G2/M. (B) Quantitative analysis representing the percentage of cells in each cell cycle phase (G0/G1, S, and G2/M) across the leukemic cell lines.

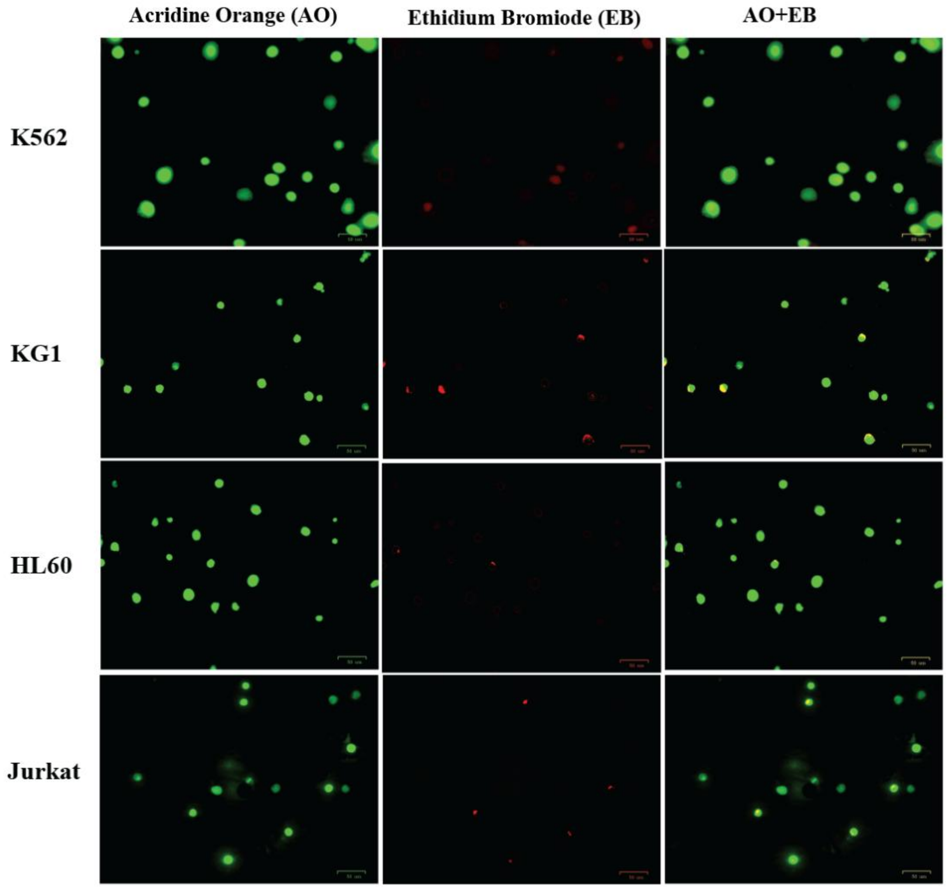

Figure S2. Assessment of apoptotic morphology in leukemic cell lines by Acridine Orange/Ethidium Bromide (AO/EB) staining. Representative fluorescence microscopic images of

K562, KG1, HL60, and Jurkat leukemic cell lines stained with Acridine Orange (AO), Ethidium Bromide (EB), and merged AO+EB staining.

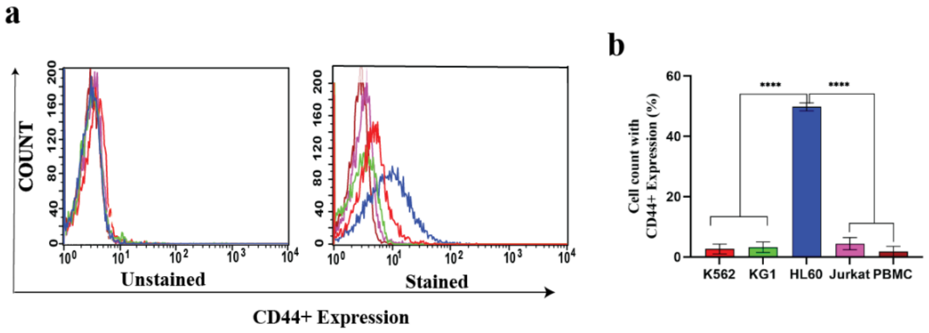

Figure S3: Flow cytometric analysis of CD44 expression in leukemic cell lines. (a) Representative flow cytometric histograms showing CD44 expression in leukemic cell lines (K562, KG1, HL60, and Jurkat) and PBMCs, comparing unstained and stained cell populations. (b) Quantitative analysis of CD44-positive cells (%) across different leukemic cell lines and PBMCs. Data are presented as mean ± SD from independent experiments. Statistical significance is indicated as ****$p < 0.0001$.
